# Psychotropic drug prescribing in an Australian specialist child and adolescent eating disorder service: a retrospective study

**DOI:** 10.1186/2050-2974-1-27

**Published:** 2013-08-08

**Authors:** Julia K Moore, Hunna J Watson, Emily Harper, Julie McCormack, Thinh Nguyen

**Affiliations:** 1 Princess Margaret Hospital for Children, Perth, Australia; 2 Centre for Clinical Interventions, Perth, Australia; 3 The School of Paediatrics and Child Health, University of Western Australia, Perth, Australia; 4 The School of Psychology and Speech Pathology, Curtin University, Perth, Australia; 5 The School of Psychiatry and Clinical Neurosciences, University of Western Australia, Perth, Australia

**Keywords:** Adolescent, Adverse effects, Binge eating, Child, Drug therapy, Eating disorders, Pharmacology

## Abstract

**Background:**

To describe the rates, indications, and adverse effects of psychotropic drug prescription in a specialist tertiary hospital child and adolescent eating disorder service.

**Methods:**

Retrospective case note study of all active eating disorder patients (*N* = 115) over the period of treatment from referral to time of study (*M* = 2 years), covering patient demographics, clinical characteristics, drug prescriptions, indications, and adverse effects.

**Results:**

Psychotropic drugs were prescribed in 45% of cases, most commonly antidepressants (41%), followed by anxiolytics (29%) and antipsychotics (22%), with 8% initiated before referral to the specialist eating disorder program. Common indications were depressed mood, agitation, anxiety, and insomnia. Patient clinical severity and complexity was associated with prescribing. Adverse effects, mostly minor, were recorded in 23% of antidepressant prescriptions, 39% of antipsychotic prescriptions, and 13% of anxiolytic prescriptions. Second generation antipsychotic prescription was associated with subsequent new onset binge eating, in this preliminary observational study. Self-harm by overdose of psychotropics occurred in 11% of patients prescribed medication.

**Conclusions:**

Psychotropic medications were frequently prescribed to adolescent eating disorder patients to treat distressing symptoms. Prospective randomised controlled trials to clarify efficacy and safety are needed. Given the difficulties of conducting clinical trials in this population, services are encouraged to monitor and audit medication safety and efficacy in everyday practice, and to report their findings.

## Background

Eating disorders (EDs) affect up to 15% of women in their lifetime [1] and have their peak incidence in adolescence [2]. The nutritional compromise, particularly in anorexia nervosa (AN), occurs during critical brain development and is associated with brain mass reduction [3] and neuropsychological abnormalities [4]. Adolescent EDs are associated with a mortality of approximately 2.9% over 6 years [5] and profound negative impacts on physical, psychological, and social development [2].

Guidelines for treatment, based on expert consensus and weak to moderate levels of evidence, recommend a specialist multidisciplinary team approach with medical, nutritional, social, and psychological care, including family and/or individual psychotherapy [6-8]. Family-based treatment, a specific treatment modality that directly addresses the ED, has a moderate level of evidence in paediatric AN [2]. There is limited evidence to guide treatment selection among paediatric bulimia nervosa (BN) or EDs not otherwise specified (EDNOS) [6,8]. Medication is not recommended as the sole treatment for any ED [6-9]. Nonetheless, psychotropic drugs are prescribed, often off-label, for comorbidities, distress, agitation, and sometimes in an attempt to treat core ED symptoms. Impact on growth and development is largely unknown, and there is little evidence to guide dosage [10].

Selective serotonin reuptake inhibitors (SSRIs) such as fluoxetine have been prescribed to adolescents with EDs for comorbid depression, anxiety, and obsessive-compulsive disorder (OCD). In young people without EDs, there is high-quality evidence supporting efficacy of fluoxetine and some other SSRIs for these disorders [11]. However, there is no evidence to support treatment with SSRIs in patients with a low body mass index; the available case reports and studies suggest that SSRIs are ineffective for symptoms of depression, anxiety, or OCD in very underweight patients, which is pertinent to AN [10]. There was no benefit from SSRIs in relapse prevention, core ED symptoms, depressive or obsessive-compulsive symptoms in a retrospective study of partially weight-restored female adolescents with AN [12]. Likewise, fluoxetine did not prevent relapse in weight-restored AN adults receiving cognitive-behavioural therapy [13]. The well-publicised finding of a small but significant increased risk of suicidality in youth after commencement of SSRIs was derived from meta-analyses of trials conducted for depression and anxiety disorders [14]; it is unknown to what extent these findings generalise to EDs. Individuals with EDs have an elevated risk of suicidality and deliberate self-harm [15,16]. Drug overdose is a leading method of suicide particularly among females [17], yet little is known about risk of overdose of prescription drugs in this context.

Second generation antipsychotics (SGAs) such as olanzapine are used as an adjunctive treatment for EDs, for anxiety, agitation, and ruminations on themes of food and body image, and to attempt to promote weight gain in malnourished patients. There has recently been interest in olanzapine treatment for AN, accompanied by increasing caution about potential adverse effects. Studies in adults with AN are mixed, though some trials suggest that SGAs improve core symptoms [18]. Prescribing pharmacotherapy involves weighing up potential benefits and risks. Norris et al. [19] published a retrospective matched-groups comparison of 86 female adolescents with AN, 43 treated with olanzapine, and 43 matched comparators. No conclusions could be drawn regarding efficacy. The most notable finding was of adverse effects in 56% of patients treated with olanzapine, most commonly sedation (40%) and lipid abnormality (29% of those screened), highlighting the need to maintain surveillance of medication safety – most older studies do not report lipid or blood glucose monitoring. Olanzapine, quetiapine, and risperidone can cause weight gain and metabolic adverse effects, such as impaired glucose tolerance and dyslipidaemia in children and adolescents, to a greater extent than in adults [20]. They may have a negative effect on bone density, mediated by hyperprolactinaemia [21]. There is also a potential for cardiac side-effects such as QT_c_ prolongation associated with SGA usage. The QT_c_ interval has been shown to be longer in AN than in healthy controls, suggesting particular vulnerability [22]. There is a case report of QT_c_ prolongation occurring repeatedly in an adolescent with AN treated with olanzapine, then risperidone, in combination with fluoxetine [23]. The cardiac risk is of particular concern for patients with AN due to nutritional deficiencies, impaired calcium and phosphate metabolism, and hypokalaemia in patients with symptoms of purging, which predispose to life-threatening arrhythmias. Hypokalaemia can cause U wave formation and flattened T waves on the ECG, which complicates the measurement of the QT_c_ interval, and so the QT_c_ interval needs to be interpreted carefully in such cases [24].

SGAs are known to increase appetite and cause weight gain, and some case reports and series, including one on a child with AN, have linked SGAs to emergence, exacerbation, and recurrence of binge eating [25-30]. McKnight and Park [31] found no published evidence that SGAs contribute to the development of binge eating, but reported their clinical observation that SGAs appear to exacerbate binge eating in patients with pre-existing bulimic symptoms. In a consecutive case series of 74 adolescent and adult psychiatric patients treated with clozapine or olanzapine, emergent binge-eating symptomatology or full-blown EDs were more common among those with a history of EDs than those without [29]. Emergent binge eating in patients with restricting AN is a common event: more than half of a sample of young adult women with restricting AN crossed over to the binge eating/purging subtype or to BN in a 7 year longitudinal study [32]. Given these findings, case reports and clinical impressions are difficult to interpret. Dysregulation of eating behaviours through either restricting or binge eating is distressing and problematic, so it is important to be vigilant regarding binge eating as a potential adverse effect of medication.

Anxiolytic drugs such as benzodiazepines are prescribed to patients with EDs to control insomnia, agitation, and anxiety and to facilitate nutritional resuscitation, but there are currently no randomized controlled trials and little literature of any kind describing their use [18]. In children with anxiety disorders, problems with disinhibition, irritability, and drowsiness have been reported with clonazepam [33]. In adults with anxiety disorders, benzodiazepines may impair memory and learning and in some instances reduce the efficacy of specific psychotherapies [34].

A recent report of psychotropic medication use in American adult women with anorexia nervosa between 1997 and 2009 found that of 539 patients presenting for bone density screening, 53% were currently taking a psychotropic medication, most commonly antidepressants (48%) and antipsychotics (13%) [21]. The use of atypical antipsychotics (most commonly quetiapine) had doubled over the 13 year period of the study. Widespread use of these medications is of concern, given the absence of conclusive evidence of benefit and the potential adverse effects.

Few studies report actual psychotropic prescribing in specialist child and adolescent ED services. In a retrospective case note study of 60 children and adolescents with AN newly referred to an Italian specialist psychiatric unit between 2000 and 2004, 16 (32%) were prescribed psychotropic medication [35]. Seventy-six percent of prescriptions were for antidepressants, most commonly paroxetine and sertraline, prescribed for comorbid depression or OCD. Antipsychotic medications accounted for 21% of prescriptions, most commonly haloperidol and olanzapine, for indications of “anguish” and “thought disorders”. Sixteen percent received two or more medications concurrently. Adverse events were reported for 29% of prescriptions. In a retrospective case note study of 308 ED cases seen in seven specialist UK child and adolescent ED services during 2005 to 2006 [36], psychotropic drugs were prescribed for 27% of patients, mainly those with AN. Fluoxetine and olanzapine were the most common prescriptions, and the most common indications were depression, anxiety and “pseudo-psychotic” concerns about weight. Adverse effects were reported for 15% of antidepressant prescriptions and 28% of antipsychotic prescriptions, but 38% of olanzapine prescriptions. No conclusions could be drawn about the effectiveness of medications.

Clinicians treating children and adolescents with EDs currently lack evidence-based guidance about psychopharmacological treatment, including efficacy, appropriate doses, and adverse effects. Little is known about how clinicians apply their experience and expert opinion. This study aimed to report the use of psychotropic drugs in a specialist setting, including medication doses and indications; to identify adverse effects; and to describe characteristics of the patient population including illness severity and comorbidity and to correlate these with prescribing practices. We also aimed to report monitoring for medication safety, including ECG monitoring and QT_c_ prolongation, and metabolic monitoring; and to document the frequency of significant adverse events such as deliberate overdose, emergent suicidality while prescribed SSRIs, and drug-related hyponatraemia. We hypothesised in general that psychotropic prescription would be associated with greater patient clinical severity and complexity, characterised by more severe eating, depressive, anxiety, and dissociative symptomatology, Axis I comorbidity, onset of deliberate self-harm acts and suicidal ideation, presence of binge eating, and admissions and bed days. Based on clinical observations, we tentatively hypothesised that there would be an association between antipsychotic use and emergent binge eating.

## Methods

### Participants

Participants were patients of Princess Margaret Hospital for Children (PMH) Eating Disorders Program (EDP). This is the only public specialist child and adolescent ED service in Western Australia (census population 2 331 500) [37]. The program offers outpatient and day-patient treatment, and inpatient treatment for medical stabilisation. The EDP accepts referrals of patients under age 16 and cares for patients up to gradual transition to adult services after age 18. At the study enrolment date, 14 April 2011, the EDP had 130 patients. At any given time, about eight EDP patients are in hospital for medical stabilisation and nutritional rehabilitation.

All ‘active’ patients of PMH EDP (*N* = 115), who met Diagnostic and Statistical Manual (DSM-IV) [38] criteria for an ED at initial assessment, were included. ‘Active’ refers to receiving treatment or follow-up of any kind at the study enrolment date. From the initial participant pool of 130, 7 patients were excluded who had not engaged following initial assessment and 8 patients were excluded as they did not meet criteria for an ED (generally had subthreshold eating problems).

### Service model and provision

All patients are initially assessed through a comprehensive multidisciplinary process, including psychosocial diagnostic interview; paediatric medical and dietetic assessment; separate interviews of child and parents using child and parent informant versions of the Eating Disorder Examination (EDE) [39]; the Children’s Depression Inventory (CDI) [40]; the Multidimensional Anxiety Scale for Children (MASC) [41]; and the Adolescent Dissociative Experiences Scale (A-DES) [42] self-report instruments. Information from the assessment is then reviewed in a multidisciplinary meeting, including the team psychiatrists, to determine a DSM-IV [38] ED diagnosis, any comorbid DSM-IV psychiatric diagnoses, and treatment plan. Further review by a team psychiatrist is readily available as needed, for example for diagnostic clarification, management planning, treatment of comorbid psychiatric disorders, and initiation and monitoring of psychotropic medication where necessary.

Patients are treated in a multidisciplinary team model, and each patient and family have a care coordinator. All patients are offered psychological therapy, including individual, group, and family therapy in various modalities. Weight restoration and physical monitoring are prioritised. Inpatient admissions for malnutrition are under the care of a paediatric gastroenterology team, with psychological medicine support, on a paediatric medicine ward, not a psychiatric ward. The goal of admission is to provide rapid, intensive nutritional rehabilitation to facilitate prompt return to family and community, often using nasogastric tube feeding. On occasion, provided that he or she is not at acute nutritional risk, a patient is admitted to a separate acute psychiatric ward for management of comorbid conditions and self-harm behaviours; close liaison is maintained with the ward clinical team regarding such patients.

The prescription of psychotropic medication usually follows psychiatric assessment by the EDP team consultant psychiatrist, psychiatry registrar or supervised medical officer. Occasionally, medications are initiated by paediatric staff. Some patients are referred already on medication prescribed by their general practitioner or psychiatrist. Within the EDP, all prescriptions are through a public hospital pharmacy at the same relatively low cost per medication per month, regardless of the medication or dosage, and further reduced for concession card holders. Cost to the patient is therefore not a major influence on prescribing practice.

### Measures

#### Eating disorder examination

Child and parent informant versions of the clinician-administered structured EDE [39], widely considered the gold standard for assessing eating disorders, measured severity of eating disorder psychopathology based on the global score. Cronbach alpha reliability in the present study was 0.94.

#### Children’s depression inventory

The 27-item CDI [40] is a well-established self-report measure of depressive symptoms with excellent reliability and construct validity, and has discriminant validity as demonstrated by evidence of its utility as a screening measure for depressive disorders [43]. Cronbach alpha reliability was 0.91.

#### Multidimensional anxiety scale for children

Anxiety symptoms were assessed with the self-report 39-item MASC, which has excellent psychometric properties [41]. Cronbach alpha reliability was 0.91.

#### Adolescent dissociative experiences scale

Dissociative symptoms were measured with the self-report 30-item A-DES, which has good reliability and validity, with increased scores corresponding to lifetime history of trauma [42]. Cronbach alpha reliability was 0.99.

#### Other sociodemographic and clinical features

These were extracted from psychological and medical records or an available clinical audit database. Children had been weighed wearing underwear and a hospital gown, using electronic scales accurate to 50 g. Height had been measured with the patient standing without shoes and socks, standing on a hard surface and using a Harpenden stadiometer, accurate to 1 mm. BMI *z*-scores had been calculated via Epi Info 7 [44] by entering the patient’s age, height, and weight into the program. *Z*-scores were age- and sex-specific based on the Centers for Disease Control and Prevention [45,46] growth charts.

### Procedure

The study received approval from the PMH Human Research Ethics Committee as a quality of care audit. A purpose designed paper form was used to collect information retrospectively from the medical file and psychiatric file of each patient, by reading through all case notes, correspondence, assessment materials, and medication charts. To test the clarity and ease of use of the form and to evaluate the inter-rater agreement for extracted data, JKM and EH both collected data from the same five randomly selected files; no discrepancies were found, yielding very high inter-rater reliability. Data collected included age, gender, date of assessment, duration of illness before referral, inpatient admissions, medical conditions, body mass index *z* score, history of self-harm or suicidal ideation or acts, binge eating history, EDE, CDI, MASC and A-DES scores; all of these are consistently and routinely recorded in a standardised assessment document for each new patient assessed by EDP. Further data collected included ECG, blood lipids, fasting blood glucose, and any extrapyramidal symptoms, hyponatraemia or QT_c_ prolongation; these were documented for some patients but not for others. Information collected about medication included prescriber, dose range, and total days of exposure, all of which were clearly documented for each patient; and indication, adverse effects, and reason for cessation, which were documented with a variable level of detail. Drug prescriptions by any prescriber within or external to the EDP were recorded, both prior to intake assessment at EDP, and subsequent to assessment, up until the date of the audit. PRN (pro re nata or “as required”) medication for inpatients was not recorded. A hospital pharmacy database of all outpatient prescriptions was used to cross-check, but no missed medications were found.

Data were anonymised at collection with a study ID. The data collection team included a psychiatrist, advanced trainee in psychiatry, clinical psychologist, research psychologist, and mental health nurses, who all met to discuss standardisation of the data collection procedure. JKM (an advanced trainee in psychiatry) designed the coding procedure, performed all coding and entered all data into a spreadsheet. All data were re-checked against the original paper forms by JKM.

### Statistical analysis

A series of analyses of variance (ANOVAs) for continuous outcomes and chi square tests of independence for categorical outcomes compared those prescribed (pre-referral and within EDP) versus not prescribed antidepressants, antipsychotics, and anxiolytics, respectively on a range of clinical characteristics across pre-referral, intake, and during service engagement. The ‘other’ psychotropic category was excluded from comparisons because of low frequency of use. The relationship between number of prescriptions and clinical characteristics was examined by way of ANOVAs and chi square testing. A logistic regression tested whether new binge onset (0 = no, 1 = yes) could be predicted from antipsychotic medication status (0 = no/prescribed antipsychotic after date of new binge onset, 1 = yes). All analyses were preliminary and exploratory, hence univariate analysis with no adjustment for multiple comparisons was used. Alpha was set at the conventional level of .05.

## Results

### Demographic characteristics

The sample included 115 patients, age 7 to 16 years, with 11 males and 104 females (90% female). The mean age at referral was 13 years (*SD* = 1.60). Average duration of engagement from referral up to the point of audit was 2 years (*SD* = 1.52 years; range = 25 days to 7 yrs 1 month).

### Clinical characteristics

At intake, most met criteria for AN (*n* = 55, 48%; restricting subtype, *n* = 48, 42%; binge-purge subtype, *n* = 7, 6%) or EDNOS (*n* = 54, 47%), and a minority had BN (*n* = 6, 5%; purging subtype, *n* = 4, 3%; non-purging subtype, *n* = 2, 2%). The mean age of onset was 12 years (*SD* = 1.66) and the mean untreated duration of illness was 9 months (*SD* = 7.73). Forty-eight percent (*n* = 55) had a comorbid psychiatric diagnosis, most commonly an anxiety disorder (27%, *n* = 31) or major depressive episode (26%, *n* = 30). Two or more comorbid psychiatric diagnoses were present in 17% (*n* = 20). Twenty-seven percent (*n* = 31) had a comorbid medical diagnosis, the most frequent being asthma/atopic syndromes.

Psychometric assessments at intake showed above average depressive and anxiety symptoms, indicated by mean *t* scores of 56 (*SD* = 21.12, *n* = 109) on the CDI and 54 (*SD* = 13.22, *n* = 108) on the MASC. Using available data, the proportion with a *t* score ≥ 65 indicating clinically significant levels was 37% on the CDI and 24% on the MASC. Lifetime suicidal ideation was reported by 28% of cases (present, *n* = 32; absent, *n* = 74, 64%; unknown, *n* = 9, 8%). Lifetime deliberate self-harm was also common (present, *n* = 18, 16%; absent, *n* = 88, 77%; unknown, *n* = 9, 8%). Three cases reported a previous suicide attempt. During engagement with the ED service, a further 8% (*n* = 8) had new onset suicidal ideation or intent, 5% (*n* = 6) had new onset suicide attempts, and 16% (*n* = 19) had new onset deliberate self-harm. Sixty-five individuals (57%) had received inpatient treatment. The median number of admissions for the full sample of 115 patients was one (range 0 to 41) and median total bed days throughout service engagement was 15 (range 0 to 921).

### Drug prescriptions

Psychotropic medication was prescribed to 52 of 115 patients (45%) pre-assessment or during engagement with PMH EDP. The youngest age at prescription was 10 years. Prescriptions were relatively infrequent for preadolescents compared to adolescents. The proportion of patients who were prescribed a psychotropic, by diagnosis, was 31/55 (56%) for AN, 4/6 (67%) for BN, and 17/54 (31%) for EDNOS.

#### Prescription frequencies prior to specialist service referral

Prior to referral, 9 cases (8%) were prescribed psychotropic medication. There were nine prescriptions, eight for antidepressants and one for lithium.

#### Prescription frequencies within the specialist eating disorder service

Forty-eight of the 115 patients (42%) were prescribed a new psychotropic medication during treatment within EDP. There were 138 prescriptions as some cases were prescribed multiple medications concurrently and/or sequentially. During engagement with EDP, 42 (37%) were prescribed antidepressants, 33 (29%) were prescribed anxiolytics or sedative/hypnotics, 25 (22%) were prescribed antipsychotics, and two (2%) were prescribed other psychotropic medications. Prescriptions were for 21 different psychotropics. The median time between assessment and prescription of psychotropic medication was 167 days (range 3 to 2012 days).

### Medication classes

Antidepressants were the most common class prescribed either pre-referral or within the ED service. Overall (pre-referral and within EDP), antidepressants were prescribed in 47 of 115 patients (41%), followed by anxiolytics in 33 (29%), antipsychotics in 25 (22%), and other psychotropics in three (3%). The main indication for antidepressants was depression (Table 1).

**Table 1:** Antidepressant prescriptions and indications in a specialist child and adolescent eating disorders program.

	**Number of prescriptions**		**Indications**											
**Dosage/day** ***(M*, range)**	**Pre-referral**	**During EDP**	**Depression mood**	**Depression & anxiety**	**Depression & OCD anxiety**	**OCD**	**Anxiety**	**Depression & OCD**	**OCD & anxiety**	**Depression & PTSD**	**Depression & insomnia**	**Anxiety & bulimic symptoms**	**Depression & bulimic symptoms**	**Unclear**
Amitriptyline (25 mg, NA)	-	1	-	-	-	-	-	-	-	-	1	-	-	-
Citalopram (32 mg, 20-60 mg)	-	6	3	2	-	-	-	1	-	-	-	-	-	-
Desvenlafaxine (83 mg, 50-100 mg)	-	3	3	-	-	-	-	-	-	-	-	-	-	-
Escitalopram (15 mg, 10-20 mg)	1	1	-	1	-	1	-	-	-	-	-	-	-	-
Fluoxetine (32 mg, 10-60 mg)	4	35	21	4	3	2	2	1	2	2	-	1	1	-
Fluvoxamine (150 mg, NA)	1	-	-	-	1	-	-	-	-	-	-	-	-	-
Mirtazapine (22 mg, 15-30 mg)	1	1	-	-	-	-	-	-	-	-	1	-	-	1
Moclobemide (150 mg, NA)	-	1	1	-	-	-	-	-	-	-	-	-	-	-
Sertraline (100 mg, NA)	1	-	1	-	-	-	-	-	-	-	-	-	-	-
Venlafaxine (168 mg, 150-225 mg)	-	4	2	1	1	-	-	-	-	-	-	-	-	-
Total	60		31 (52%)	8 (13%)	5 (8%)	3 (5%)	2 (3%)	2 (3%)	2 (3%)	2 (3%)	2 (3%)	1 (2%)	1 (2%)	1 (2%)

### Antidepressants

Fluoxetine was the most frequently prescribed antidepressant (see Table 1 for indication and dosage characteristics), comprising 39/60 (65%) of antidepressant prescriptions. Adverse effects were reported for 9/39 (23%) prescriptions. Although most were minor (sedation, dizziness, tremor), there was one case of new onset deliberate self-harm, two cases of small intentional overdose, and one case of large intentional overdose. (Here, and in all instances that follow, “small intentional overdose” is used when the admitting emergency department doctor described the overdose as “small” and no treatment or monitoring was required; “large intentional overdose” is used when the admitting doctor described the overdose as “large” or inpatient medical treatment, cardiac or respiratory monitoring, pharmacological antidote, or intensive care was given). Fluoxetine was ceased in 15 instances, due primarily to either ineffectiveness or patient disengagement.

Twenty-one of 60 (35%) antidepressant prescriptions were for medications other than fluoxetine (see Table 1). Many prescriptions (38%, 8/21) occurred after a trial of fluoxetine. Five patients prescribed other antidepressant medications had adverse effects. A patient prescribed citalopram took a large overdose, while serotonin syndrome and new onset of suicidality and deliberate self-harm were noted in a patient prescribed moclobemide. One of the two patients prescribed mirtazapine developed new onset binge eating. Dizziness was reported with one patient prescribed sertraline. Medication was ceased in 8/21 instances (38%).

There was one case of hyponatraemia (plasma sodium 115 mmol/L) associated with fluoxetine treatment in an underweight 16 year-old male who had excessive water consumption; plasma sodium normalised with normalisation of water intake, while fluoxetine treatment continued.

### Antipsychotics

The main indications for antipsychotic medications were agitation and anxiety (Table 2). All prescriptions were made during EDP treatment. There were 32 prescriptions for antipsychotics, 24/32 for quetiapine, 7/32 for olanzapine, and 1/32 for risperidone. Adverse events were reported in 41% (13/32) of prescription instances. Among these were nine cases prescribed quetiapine; one took a large overdose, there was clinical concern about precipitation or exacerbation of binge eating in two cases, one had extrapyramidal side effects while taking quetiapine 75 mg in combination with fluoxetine 20 mg and lorazepam 3 mg, one had QT_c_ prolongation, three reported somnolence, and one reported nightmares. In four cases there was clinical concern that olanzapine may have precipitated or exacerbated binge eating. Antipsychotic medication was ceased in 69% (22/32) of instances.

**Table 2:** Antipsychotic prescriptions and indications in a specialist child and adolescent eating disorders program.

	**Number of prescriptions**		**Indications**											
**Dosage/day** ***(M*, range)**	**Pre-referral**	**During EDP**	**Anxiety**	**Agitation**	**Anxiety & agitation**	**Anxiety & insomnia**	**Depression & anxiety**	**Depression & PTSD**	**Depression & agitation**	**PTSD**	**Depression**	**Depression & insomnia**	**Depression & insomnia & affective dysregulation**	**OCD & anorexic ruminations**
Olanzapine (6.8 mg, 2.5-15 mg)	-	7	2	3	1	-	1	-	-	-	-	-	-	-
Quetiapine (56 mg, 12.5-200 mg)	-	24	7	4	3	2	1	1	1	1	1	1	1	1
Risperidone (1 mg, NA)	-	1	-	1	-	-	-	-	-	-	-	-	-	-
Total	32		9 (27%)	8 (24%)	4 (12%)	2 (6%)	2 (6%)	1 (3%)	1 (3%)	1 (3%)	1 (3%)	1 (3%)	1 (3%)	1 (3%)

### Anxiolytics and sedative-hypnotics

These were prescribed primarily for insomnia, anxiety, and agitation (Table 3). Lorazepam comprised more than half (28/46) of these prescriptions. Adverse effects were experienced by 13% (6/46) of those prescribed anxiolytics, all of whom were taking lorazepam. One adolescent took a small overdose, four reported somnolence, and one reported nausea. Medication was ceased in 83% of prescription instances (38/46).

**Table 3:** Anxiolytic prescriptions and indications in a specialist child and adolescent eating disorders program.

	**Number of prescriptions**		**Indications**							
**Dosage/day (*M*, range)**	**Pre-referral**	**During EDP**	**Insomnia**	**Anxiety**	**Anxiety & agitation**	**Agitation**	**Anxiety & insomnia**	**Depression & PTSD**	**PTSD**	**Unclear**
Clonazepam (2.3 mg, 1–4 mg)	-	4	-	-	2	1	1	-	-	-
Lorazepam (1.7 mg, 0.5-4 mg)	-	28	2	12	4	4	2	1	1	2
Melatonin (1.9 mg, 1.5-2 mg)	-	5	5	-	-	-	-	-	-	-
Nitrazepam (15 mg, 10–20 mg)	-	2	2	-	-	-	-	-	-	-
Temazepam (10 mg, 5–20 mg)	-	7	6	-	-	-	-	-	-	1
Total	46		15 (33%)	12 (26%)	6 (13%)	5 (11%)	3 (7%)	1 (2%)	1 (2%)	3 (7%)

### Other medications

One patient was prescribed lithium pre-referral for a diagnosis of bipolar disorder. Lithium was ceased after EDP assessment with revision of the diagnosis. During EDP engagement, one patient was prescribed St John’s wort by an external clinician for depression, and one was prescribed atomoxetine then dexamphetamine for attention deficit-hyperactivity disorder.

### Pharmacotherapy vs. non-pharmacotherapy patient characteristics

Exploratory ANOVAs and chi square tests comparing those prescribed and not prescribed antidepressants on the clinical variables in Table 4 (results not presented), showed that the group prescribed antidepressant medication had significantly higher EDE, CDI, A-DES, Axis I comorbidity, binge eating presence, admissions, bed days, new onset deliberate self-harm and suicidality, and were less likely to have EDNOS (*p*s < .05). The group prescribed antipsychotic medication had significantly higher EDE, CDI, Axis I comorbidity, admissions, bed days, binge eating presence, and new onset deliberate self-harm and suicidality. Compared to those who did not receive anxiolytics, the group prescribed anxiolytics had higher EDE, CDI, Axis I comorbidity, admissions, bed days, binge eating presence, and new onset deliberate self-harm and suicidality, and were less likely to have EDNOS (*p*s < .05).

**Table 4:** Pre-referral, intake, and during service engagement characteristics of those prescribed none, one to two, or three or more psychotropic medications within a specialist child and adolescent eating disorders program.

		**Number of prescriptions**				
**Time**	**Characteristic**	**0 (*n*** **= 66)**	**1-2 (*n*** **= 27)**	**3+ (*n*** **= 22)**	***p***	**post hoc**
Pre-referral	Age at onset, yrs	12.79 (1.79)	12.81 (1.64)	12.95 (1.29)	ns	-
	Untreated duration of illness, mths	8.64 (7.61)	11.11 (7.98)	8.89 (7.76)	ns	-
	History of DSH/suicidal ideation	27% (16)	33% (8)	36% (8)	ns	-
	History of DSH acts	15% (9)	21% (5)	22% (4)	ns	-
	History of suicidal acts	3% (2)	0% (0)	5% (1)	ns	-
Intake	Age at referral, yrs	13.50 (1.76)	13.85 (1.49)	13.73 (1.60)	ns	-
	Primary diagnosis				ns	-
	AN	41% (27)	48% (13)	68% (15)	-	-
	non-AN	59% (39)	52% (14)	32% (7)	-	-
	(BN)	3% (2)	7% (2)	9% (2)	-	-
	(EDNOS)	56% (37)	44% (12)	23% (5)	-	-
	Axis I comorbidity	29% (19)	74% (20)	73% (16)	<.001	0 < exp; 1–2 > exp
	EDE	2.53 (1.57)	2.94 (1.59)	3.88 (1.58)	.003	1-2, 3+ > 0
	CDI *t* score	51.60 (21.87)	59.77 (17.80)	67.045 (18.33)	.008	3+ > 0
	MASC *t* score	52.94 (13.47)	53.24 (11.66)	59.10 (13.22)	ns	-
	ADES	1.95 (1.71)	2.16 (1.80)	2.57 (2.17)	ns	-
	BMI *z* score	−1.50 (1.38)	−1.60 (1.20)	−1.76 (.96)	ns	-
EDP	Inpatient admissions	0.48 (.71)	2.04 (2.28)	6.82 (8.82)	<.001	3+ > 0, 1-2
	Inpatient bed days	11.67 (18.59)	59.96 (82.69)	166.05 (218.58)	<.001	3+ > 0, 1-2
	DSH new onset	2% (1)	20% (5)	59% (13)	<.001	0 < exp; 3+ > exp
	Suicidality new onset	3% (2)	15% (4)	36% (8)	<.001	0 < exp; 3+ > exp
	Presence of objective binge episodes	24% (16)	38% (10)	68% (15)	.001	3+ > exp

Data are presented as means (standard deviations) or percentages (number) and are based on available data only (i.e., missing or unknown data excluded). Chi square post hoc analyses indicate whether cell counts are lower or higher than would be expected based on equivalent count distributions. ADES = Adolescent Dissociative Experiences Scale; AN = anorexia nervosa; BN = bulimia nervosa; BMI = body mass index; CDI = Children’s Depression Inventory; DOI = duration of illness; DSH = deliberate self-harm; EDE = Eating Disorder Examination; EDNOS = eating disorders not otherwise specified; EDP = eating disorders program; exp = expected cell count; MASC = Multidimensional Anxiety Scale for Children. Missing data for some individuals; available data: EDE *n* = 111, CDI *n* = 109, MASC *n* = 108, ADES *n* = 91, DSH new onset, *n* = 110.

### Number of prescriptions and polypharmacy

The total number of drugs prescribed per patient within PMH EDP ranged from 0 to 7. ANOVA and chi square analyses showed that of the clinical variables presented in Table 4, a higher number of prescriptions was associated with clinical severity (characterised by more severe eating and depressive symptomatology, Axis I comorbidity, onset of deliberate self-harm acts and suicidal ideation, presence of binge eating, and more admissions and bed days).

Concurrent prescriptions (i.e., having more than one prescription at a time) occurred during service engagement in 30% (34/115) of patients. The maximum number of concurrent prescriptions was four, with the following proportions: two drugs for 17% (19/115), three drugs for 10% (12/115), and four drugs for 3% (3/115). An antidepressant combined with an anxiolytic, an antipsychotic, or both, were the most common combinations.

### Medication safety

Safety considerations studied included QT_c_ prolongation, ECG monitoring and metabolic monitoring for SGAs, deliberate overdose, and emergent suicidality with SSRIs. Another objective was to investigate the possibility of emergent binge eating with antipsychotics.

QT_c_ prolongation on ECG of clinical concern was recorded in two patients; both were nutritionally compromised, dehydrated female adolescent inpatients. The QT_c_ prolongation did not result in adverse clinical consequences. One patient was prescribed olanzapine, clonazepam, nitrazepam and omeprazole, and had normal electrolytes. Medications were continued, and the mild QT_c_ prolongation of 469 ms resolved over three weeks as her nutritional state improved. The other was prescribed fluoxetine and recently commenced quetiapine, and had hypophosphataemia. Quetiapine was ceased, she was rehydrated and electrolytes were normalised, and the QTc prolongation of 500 ms resolved within 24 hours. Quetiapine was never reintroduced.

Only four of the 25 patients treated with a SGA had an ECG at baseline, and two had a repeat ECG within 2 weeks of initiation.

Self-harm by overdose was observed in 11% (6/52) of patients prescribed a psychotropic drug, with one taking an overdose on two separate occasions. Although there were no permanent sequelae, three instances involved large, potentially dangerous dosages.

In patients prescribed SGAs, none had monitoring of blood glucose or lipids at any time.

There were no completed suicides in the cohort (or in previous active cases since program inception). Of 106 evaluable patients, 3/42 (7%) prescribed antidepressants with an FDA black box warning label of suicidal risk (fluoxetine, sertraline, paroxetine, citalopram, escitalopram, fluvoxamine) experienced suicidality onset within 3 months of initiation, and overall 14/106 (13%) had suicidality onset at some time.

Eighty-three percent (96/115) of patients were binge abstinent at intake, and 19% (22/115) of the entire sample developed new binge eating during treatment in the EDP. An exploratory logistic regression showed that medication status reliably distinguished between those with and without new binge onset, χ^2^(1) = 3.93, *p* = .047. The odds ratio of new binge onset was 3.5 times as large for a patient prescribed antipsychotic medication than the odds for a patient not prescribed antipsychotic medication. Of the six patients with new binge onset after SGA initiation, three were prescribed olanzapine and three quetiapine. The exposure time before recorded binge onset ranged from 7 to 667 days (*M* = 260 days exposure, *SD* = 235 days).

## Discussion

To the best of our knowledge, this is the first retrospective case note study of psychotropic drug prescribing in an Australian specialist child and adolescent ED program. Forty-five percent of patients were prescribed psychotropic medication. In our sample, 48% had one or more comorbid Axis I psychiatric diagnoses, consistent with reported high comorbidity [47,48]. Those with psychiatric comorbidity were significantly more likely to be prescribed medication. There was a significant relationship between prescribing (including number of prescriptions) and disease severity and number and total length of hospital admissions. Adverse effects of medication were commonly recorded and somewhat higher for antipsychotic medications (39%) than antidepressants (23%). Most of these adverse effects were minor, although there were a number of instances of non-fatal self-harm by overdose of medication. Deliberate overdose of prescribed medication was relatively frequent. The study showed a very preliminary association between emergent binge eating and antipsychotic use in this population, which may be explained by mechanisms linking SGAs and appetite dysregulation [49]. Before being accepted, this finding needs replication to establish that it is not reflective of sampling variability or a Type I error.

The prevalence of psychotropic prescription in this study was higher than reported by Gowers et al. [36] or Rossi et al. [35]. It is difficult to compare services operating in different countries, which probably have different patient populations, service resources, and care philosophies. In our sample 57% received inpatient treatment at some time compared with 41% in Gowers et al. [36]. These figures are not directly comparable, as different services have different criteria for admission, but may suggest a more severely unwell population in our study. Gowers et al. also reported a prevalence of 23% for comorbid psychiatric disorders, less than half the prevalence for our sample. The study designs differed, for example, Gowers et al. reported on prescribing during a 12-month period for all subjects, whereas we reported prescribing during the full period of engagement with the EDP, which averaged 24 months.

The medication dosages used were low to medium for antipsychotics. For antidepressants, most dosages were low to medium, but a few patients were on higher doses of selective serotonin uptake inhibitors, in line with usual clinical practice for adults with bulimia nervosa. No conclusions can be drawn about effectiveness or safety of dosages from the data.

Patients within our service were prescribed more benzodiazepines, such as lorazepam, than reported by Gowers et al. [36]: 29% of total prescriptions were for benzodiazepines, often for a short period. There were no cases of benzodiazepine abuse or other serious adverse effects. Severe and persistent insomnia was a common, distressing symptom in our patient population. Insomnia was noted as a consequence of malnutrition in a classic starvation experiment [50], though clinical literature on insomnia in EDs is lacking. We speculate that the rate of benzodiazepine prescription may reflect the clinical difficulties of managing acute re-feeding on a busy general paediatric ward without specialist mental health nursing and with limited resources to provide structured support for EDP patients: anxiety, agitation, and insomnia are common complaints in this setting.

Patients with EDs constitute a highly distressed, high-risk population, with prevalent suicidal attempts and ideation and deliberate self-harm. Among adults, 3% to 20% with AN and 25% to 35% with BN report a history of attempted suicide; completed suicide is more common in AN than BN [15,16]. Exact rates among children and adolescents are unknown. In our study, 11% of patients prescribed medication (6/52 patients) self-harmed by overdose on their prescribed psychotropic medication. Attention to prescribing medications of lower risk in overdose, limiting amount dispensed, observing medication being swallowed to reduce potential for hoarding, and engaging patients and families in maintaining safe medication storage are commonsense measures.

There are no data to indicate whether suicidality risk increases with SSRIs in a paediatric ED population; however, given there is a heightened risk in youth with anxiety and depressive problems to age 25 years, and that anxiety and depressive comorbidity is common in ED populations, this is an important safety concern. Of 20 patients who developed suicidality during service engagement, three of the 18 evaluable who had onset date recorded were within three months of SSRI initiation. Routine clinical monitoring for suicidality in ED populations is imperative, and future investigation of suicidality risk with SSRIs in paediatric ED patients is warranted.

Routine monitoring of metabolic parameters in patients prescribed antipsychotic medications is recommended by Australian guidelines [51]. Hyperlipidaemia is a common complication of antipsychotic therapy even in underweight young patients with EDs [19]. The PMH protocol for SGAs specifies regular monitoring of metabolic parameters; monitoring of weight and blood pressure is universal due to the close medical monitoring of EDP patients, but clinician compliance with monitoring of blood lipids and glucose was lacking. Compliance with ECG monitoring for QT_c_ prolongation in patients prescribed SGAs was low. This problem of translating evidence-based guidelines into routine clinical care is not unique to our service; low compliance with metabolic monitoring has been reported internationally [52]. An Australian specialist youth service has recently reported on barriers and enabling factors for metabolic monitoring within a first episode psychosis clinic [52] and showed substantial improvement in screening and monitoring after a package of interventions [53].

Limitations of this study include a retrospective audit design; adverse effects of medication may be under-recorded when pharmacovigilance occurs via spontaneous reporting [54]. This is likely to be particularly true for binge eating, which tends to be concealed. The retrospective design led to limitations due to variability in documentation; for example, if a prescriber documented “anxiety” as an indication for medication, it was not always clear whether the patient met criteria for a particular DSM-IV anxiety disorder at that time. Comorbid Axis I diagnoses were assigned clinically without the administration of gold standard structured interviews, which reduces reliability. We classified deliberate overdose of prescription medication as an adverse effect related to that medication; this methodology may differ from that used by other authors. No conclusions can be drawn about efficacy of psychotropic medications in managing EDs. Our results may differ from the prescription of medication in other ED treatment settings. In particular, because all medications were supplied at standard PBS charges, these findings may not generalise to other settings in which high cost to the patient may limit off-label use of antipsychotic medications. Analyses are preliminary and exploratory, with many variables, and occurred in the context of an observational design with a sample size pre-determined by the “active” caseload; in future replications due attention will need to be given to statistical power and controlling the Type 1 error rate. Chi-square analyses must be interpreted tentatively as sometimes data were unevenly distributed across cells, leading to violations in expected cell counts. Nonetheless, the presented results correlating prescribing practices to severity and complexity of presentation have clinical validity. Adherence to prescribed medication could not be formally evaluated due to the retrospective audit design; therefore, safety and risk data may underestimate actual risk. We enrolled only patients who were “active”, that is, receiving some form of treatment or follow up at the study commencement date. To maximise current clinical relevance, we chose to study only the current group, as prescribing practices and medications available have changed over the 10 years since the EDP’s inception; however, this led to a potential source of bias by excluding patients who had dropped out of treatment, who may have different characteristics.

## Conclusions

Our hope is that this study will provide a template for future studies, which may include the follow up of this cohort, as well as multicentre collaboration examining the efficacy and utility of psychotropic medication in managing paediatric EDs, with larger samples and a prospective design. Given the difficulty of conducting large randomised controlled trials of medication in this patient population [55], reports of actual prescribing practices, medication safety, and treatment outcomes in a naturalistic setting are of value. Understanding the prevalence and indications of psychotropic use is essential to considering their place in clinical practice and implications for service provision, including treatment and safety monitoring. We propose some principles to guide prescription of psychotropics for children and adolescents with eating disorders (Table 5).

**Table 5:** Proposal for prescribing psychotropic medication for children and adolescents with eating disorders.

**Principle**	**Key elements**
1. Adjunctive treatment in selected patients	a. In context of comprehensive medical and psychological assessment and treatment, including weight restoration
	b. For relief of persistent distressing symptoms
	c. For treatment of comorbid psychiatric disorders
2. Informed consent	a. Patient and family/carers
	b. Discuss rationale, potential benefits, potential risks, alternative treatment options, data available
	c. Choice of medication informed by individual characteristics
	d. Documentation
3. Record baseline information	a. Identify target symptoms
	b. Document level of baseline symptoms and social/occupational function
	c. Use appropriate pre and post rating scales
	d. Screen for suicidality and binge eating
	e. Baseline investigations e.g. electrolytes, ECG, fasting lipids and fasting blood sugar prior to prescribing SGAs
4. Treat	a. Start at low doses, increase cautiously
	b. Safe prescription and storage to reduce harm from intentional overdose
	c. Enlist patient and family in adherence and monitoring
5. Monitor	a. Monitor treatment response clinically and using appropriate rating scales
	b. Monitor for adverse effects
	1. Screen for binge eating and suicidality
	2. Examination for extrapyramidal adverse effects of SGAs
	3. Monitor for SGAs with ECG, serum lipids, fasting blood sugar, weight, any other measures specific to the drug
6. Review	a. Regular review of balance of benefit and risk
	b. Continue if effective and tolerated, cease if ineffective or poorly tolerated
	c. Minimise polypharmacy
	d. Maintain emphasis on non-pharmacological treatments
	e. Review appropriateness as patient’s nutritional state changes
7. Audit	a. Collect and share data on prescription, effectiveness and adverse effects
	b. Implement service-wide procedures that facilitate good prescribing and monitoring practices and allow data collection

## Abbreviations

A-DES: Adolescent dissociative experiences scale; AN: Anorexia nervosa; ANOVAs: Analyses of variance; BN: Bulimia nervosa; CDI: Children’s depression inventory; DSM-IV: Diagnostic and statistical manual; ECG: Electrocardiogram; EDE: Eating disorder examination; EDs: Eating disorders; EDNOS: EDs not otherwise specified; EDP: Eating disorders program; MASC: Multidimensional anxiety scale for children; OCD: Obsessive-compulsive disorder; PMH: Princess margaret hospital for children; PRN: Pro re nata or “as required”; SGAs: Second generation antipsychotics; SSRIs: Selective serotonin reuptake inhibitors.

## Competing interests

The authors report no conflicts of interest. The authors alone are responsible for the content and writing of the paper.

## Authors’ contributions

All authors contributed to the design and implementation of the study, reviewed and edited drafts of the manuscript, and read and approved the final draft. JKM undertook the literature review, conducted a proportion of the medical record reviews, coded and entered the data, contributed to the quality control and data interpretation, contributed to the initial draft, and edited the final draft. HJW conducted a proportion of the medical reviews, conducted the quality control, data analysis and interpretation, and contributed to the initial draft. EH conducted the major proportion of medical record reviews and contributed to the initial draft. JM had a significant role in study design and data interpretation, and was involved in revising the manuscript critically. TN conducted a proportion of the medical record reviews and made particular contributions to data interpretation. All authors read and approved the final manuscript.
